# In vitro study on the osteoimmunological potential of magnesium implants (WE43MEO)

**DOI:** 10.1186/s12938-025-01413-5

**Published:** 2025-07-15

**Authors:** Sabrin Aydin, Ana Prates Soares, Heilwig Fischer, Raphael Silvan Knecht, Alexander Kopp, Katharina Schmidt-Bleek, Max Heiland, Carsten Rendenbach

**Affiliations:** 1 Department of Oral and Maxillofacial Surgery, Charité—Universitätsmedizin Berlin, Corporate Member of Freie Universität Berlin, and Humboldt-Universität Zu Berlin, and Berlin Institute of Health, Berlin, Germany; 2 Julius Wolff Institute for Biomechanics and Musculoskeletal Regeneration, Berlin Institute of Health at Charité—Universitätsmedizin Berlin, Berlin, Germany; 3 Zentrum Für Muskuloskeletale Chirurgie, Charité—Universitätsmedizin Berlin, Corporate Member of Freie Universität Berlin, and Humboldt Universität Zu Berlin, and Berlin Institute of Health, Berlin, Germany; 4 BIH Biomedical Innovation Academy, BIH Charité Clinician Scientist Program, Berlin Institute of Health at Charité —Universitätsmedizin Berlin, Berlin, Germany; 5 Meotec GmbH, Aachen, Germany; 6 Berlin Institut of Health Center for Regenerative Therpaies, Berlin Institute of Health at Charité – Universitätsmdizin Berlin, Berlin, Germany

## Abstract

**Introduction:**

Bioresorbable implants significantly advance orthopedics and regenerative medicine, offering advantages over permanent implants for bone regeneration. They eliminate the need for secondary surgery and reduce long-term risks associated with permanent implants. Magnesium-based alloys are particularly promising, as their biocompatibility and mechanical properties are similar to bone. However, the degradation of magnesium is associated with physiological challenges that need to be better understood.

**Objective:**

The primary focus of this in vitro study was to investigate the osteogenic and immunomodulatory potential of WE43, a promising magnesium alloy tailored for clinical applications, and to test its osteogenic effect when a plasma electrolytic oxidation (PEO) surface modification is added.

**Results:**

The present data revealed that WE43 implants show excellent biocompatibility and bioactivity, promoting the viability of osteoblasts and enhancing the expression of osteogenic genes, specially Alpl and Tnfrsf11b. PEO surface modification did not further enhance osteogenic differentiation. Notably, WE43 implants elicited a minimal inflammatory response in RAW264.7 murine macrophages, indicating good biocompatibility. Furthermore, supernatant collected from RAW264.7 murine macrophages cultured with WE43 implants stimulated the Alpl expression in MC3T3-E1 murine osteoblasts, demonstrating their potential osteoimmune effect.

**Conclusion:**

The present findings highlight the promising potential of WE43 alloy as a biocompatible and osteoinductive biomaterial for bone regeneration applications. Their osteoimmune modulation further demonstrates the advantages of using this alloy system. Specifically, a minimal, well-controlled inflammatory response can promote a faster transition to the bone remodeling phase, leading to quicker and more effective bone regeneration.

**Methodology:**

A comprehensive in vitro investigation was conducted to assess the impact of both WE43 and WE43 PEO on the viability, Alkaline Phosphatase (ALP) expression, osteogenic gene expression (Alpl, Tnfrsf11b, and Bglap), and mineralization of MC3T3-E1 murine osteoblasts. The osteoimmunomodulatory response to WE43 was evaluated using RAW264.7 murine macrophages by assessing their response to direct contact with the alloy.

**Supplementary Information:**

The online version contains supplementary material available at 10.1186/s12938-025-01413-5.

## Introduction

The discovery of bone implants that fixate, support healing, and degrade within the body has revolutionized the field of bone fixation. This approach centers around the idea of a biomaterial that provides temporary structural support, degrading and disappearing once the healing process is complete. Such desirable properties eliminate the need for secondary surgeries and minimize long-term risks [1]. However, the complexity of bone healing, with its intricate interplay between osteogenic and immunologic systems [2] underscores the significance of considering the osteoimmunomodulatory properties of bioresorbable materials. The immune system plays a vital role in bone regeneration, influencing the balance between bone formation and resorption [3]. An ideal biomaterial should reinforce the bone structure and actively accelerate and promote bone formation in the healing area while positively influencing the local immune response. This modulation requires a controlled inflammatory response that supports tissue repair and regeneration without excessive inflammation or implant rejection [4].

Magnesium is a promising candidate among biodegradable bone fixation biomaterials due to its unique properties. In addition to its biocompatibility, the mechanical properties of magnesium are inherently similar to those of bone as they have comparable densities and elastic moduli [5]. Moreover, magnesium naturally exists in bone composition and is essential for metabolic processes [6]. However, the rapid degradation of magnesium due to uncontrolled corrosion presents challenges. Magnesium oxidation generates gas, which can accumulate around the implant, forming gas pockets and inhibiting bone healing and implant integration [7]. Nevertheless, in vitro studies have demonstrated that magnesium ions released during the degradation have been found to stimulate osteoblast activity and bone formation, suggesting a potential benefit to bone regeneration [8]. This duality of effects underscores the urgent need for further research to fully understand the impact of magnesium and its alloys on bone healing.

Various strategies have been used to control the oxidation rate of magnesium-based biomaterials, including alloying and surface modification [9]. Different strategies are used to adjust magnesium alloys' physical and chemical properties for biomedical use. Alloying can improve the mechanical performance and oxidation rate [10]. Magnesium alloy WE43, an Mg-Y-RE-(Zr) alloying system, has successfully shown superior corrosion resistance compared to more conventional alloys like AZ31 and AZ61 [10, 11]. Another successful approach to reducing the degradation rate of magnesium is surface modification, specifically plasma electrolytic oxidation (PEO), also referred to as micro-arc oxidation (MAO) [9]. PEO is a surface treatment process using high-voltage initiated sparks in an electrolyte bath close to room temperature, creating plasma discharges on a film-forming metal surface and converting it into a hard, ceramic-like surface layer [10].

While various surface modification techniques exist, PEO offers distinct advantages for magnesium alloys in biomedical applications. PEO creates a thick, ceramic-like oxide layer, enhancing corrosion resistance compared to many traditional coating methods, and can incorporate elements to improve biocompatibility. Furthermore, PEO coatings exhibit strong adhesion and the process is relatively cost-effective [12]. These combined properties justify the use of PEO as a clinically relevant surface treatment for biodegradable magnesium implants.

The combination of WE43 with PEO has decreased degradation in vitro by more than half [10]. Furthermore, data from Rendenbach et al. [13], revealed that the presence of PEO on WE43 implants in vivo promoted better osteointegration after 6 and 12 months of implantation.

Despite these discoveries, questions remain regarding the impact of WE43, with or without PEO, on the osteogenic process, especially regarding the influence of the WE43 alloy on the early osteoimmune response. Hence, the present study aimed to compare the effect of WE43 with and without PEO surface modification on the osteogenic process using MC3T3-E1 subclone 4 osteoblasts in vitro. In addition, the present work also aims to investigate the immune response to WE43 using RAW264.7 macrophages as well as the osteoimmunological effect of WE43 in a co-culture model to complement the state-of-the-art on the osteoimmunological potential of surface-modified WE43 magnesium implants.

## Results

### Material characterization

#### SEM imaging and EDX analysis

The EDX results (Supplementary Fig. 1) showed that the WE43 samples presented a uniform surface with 100% Mg. The results also revealed that the WE43 PEO showed the presence of C (8.52%), O (26.91%), Mg (28.79%), Al (1.89%), and P (33.89%). The high amount of Oxygen can be counted for the oxidation of the surface and the presence of the other elements in an oxidized state (as shown by Kopp et al. [14]). Furthermore, the measured amount of Al seems to be associated instead with the background signal originating from the aluminum-based SEM chamber and holders, since it is nominally not present in the alloy.

**Fig. 1:**
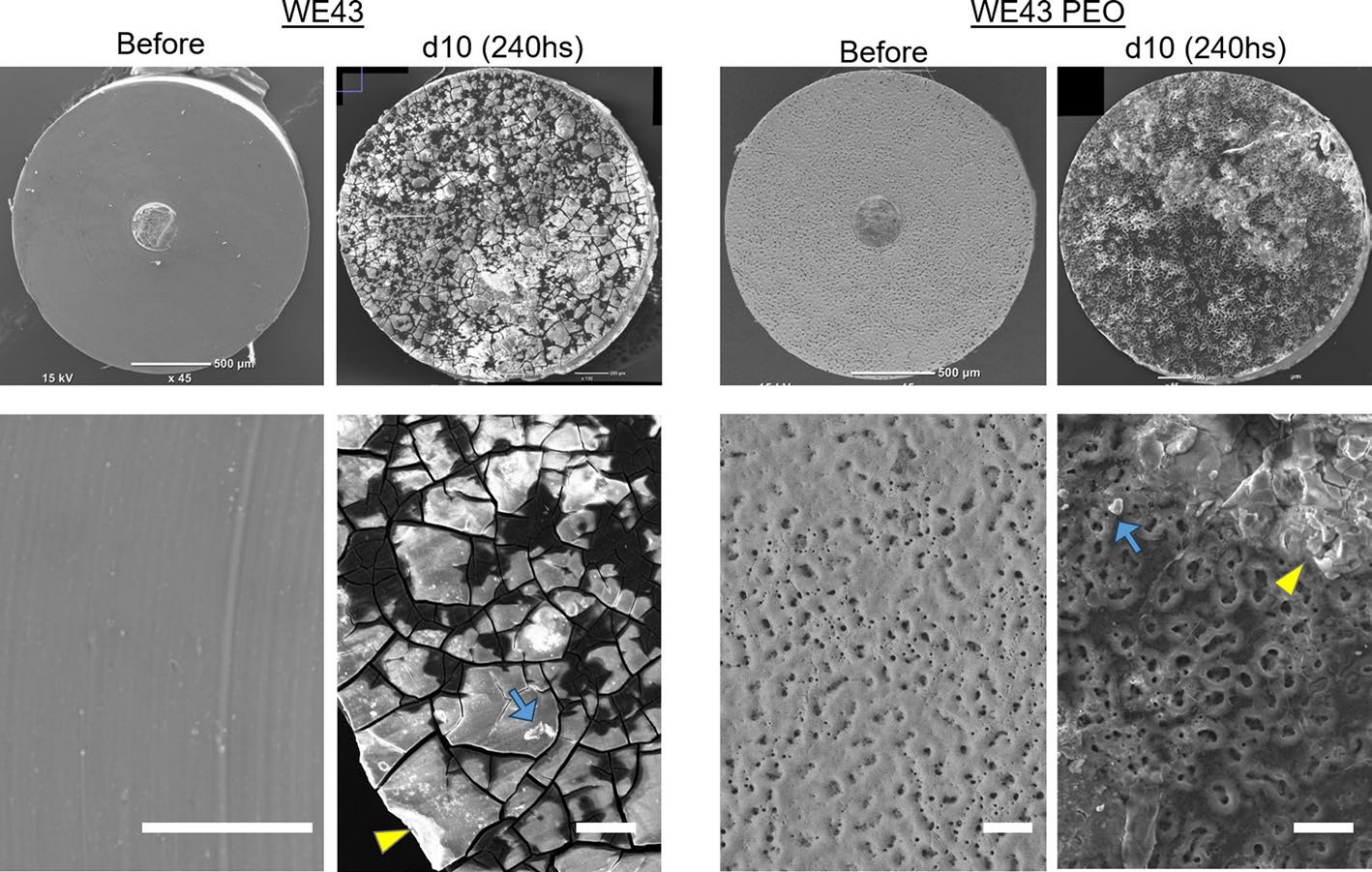
Overview of WE43 and WE43 PEO samples in culture medium before and after 10 days, revealing their surface structure presenting material detachment (yellow arrowhead) and accumulated organic matter (blue arrow) from the FBS on their surfaces. Group sizes were n = 3. Scale bar = 50 µm

Imaging the Mg samples before the experiment and after 10 days (240 h) in culture medium revealed the extent of degradation evolving on the surface (Fig. 1). The WE43 samples before the experiment presented a mostly smooth surface with visible tool marks (grooves) from the Swiss turning processing. The samples taken after 10 days of the experiment were air-dried and imaged. This air-drying step is necessary for SEM imaging to remove residual moisture. The surface of WE43 samples showed a typical degradation layer mainly containing magnesium hydroxides and forming a film. The stresses induced during air-drying can cause this film to shrink and crack. The WE43 PEO samples exhibited a typical PEO surface layer before the experiment, displaying the characteristic irregular surface formation with random pore patterns ranging from 1 to 5 µm in diameter. After 10 days, the pores' shapes were more extensive, ranging from 10 to 25 µm, likely due to the progressing degradation starting in the pore grounds and extending to the pore cavities. Moreover, after 10 days, both types of samples presented areas of material detachment and accumulated organic matter from the FBS on their surfaces. The images revealed the apparent differences in the geometry of degradation. At the same time, the non-surface-modified WE43 samples presented a passivated and degraded surface. In contrast, the PEO samples presented a generally more intact appearance after the testing period, revealing the extent of degradation in a more pronounced surface porosity rather than an overall corrosion attack on the surface.

#### Implant degradation

**Fig. 2:**
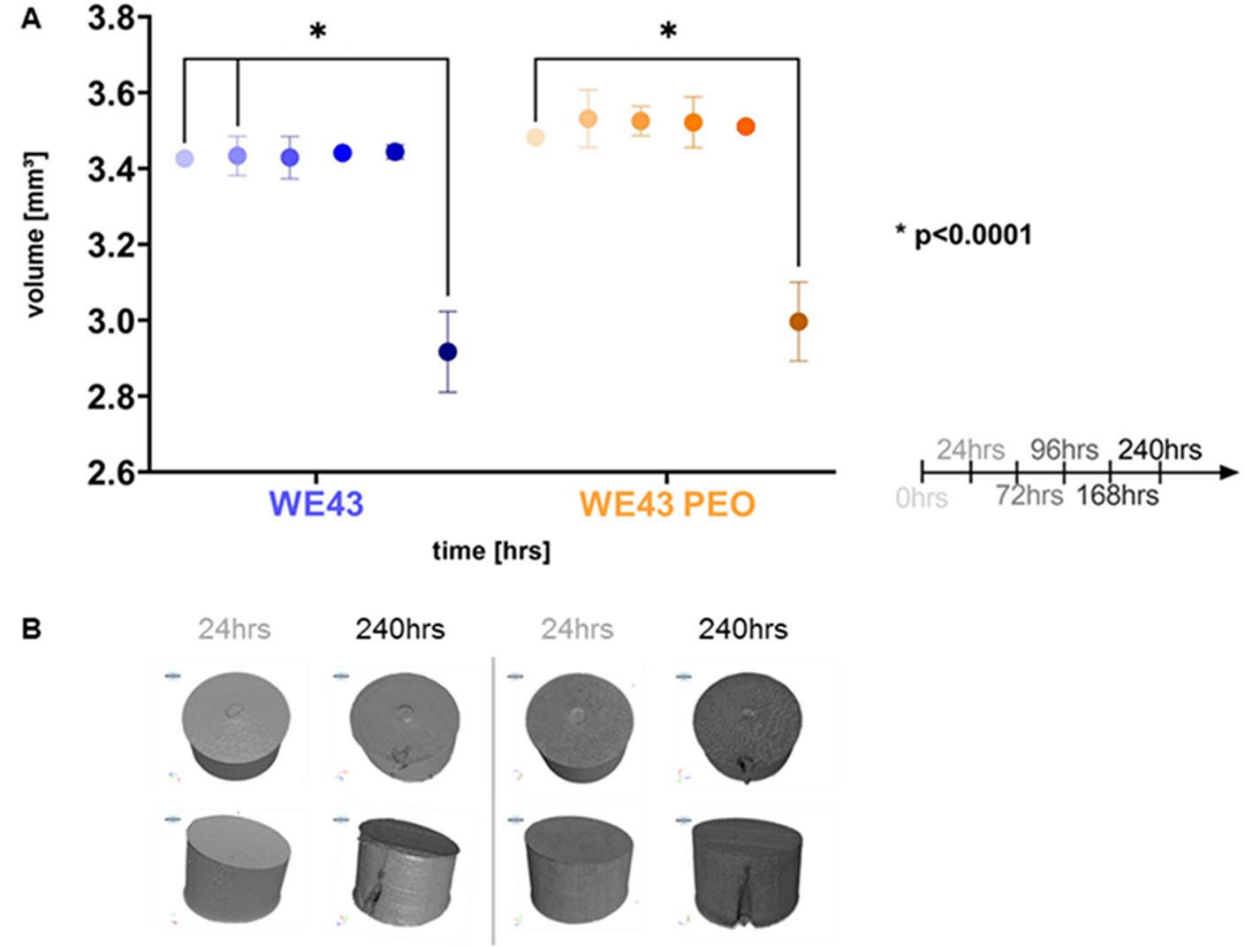
Degradation of the Magnesium samples. **A** Graph with sample volume vs. time: Statistical differences are analyzed by two-way ANOVA. The error bars represent mean ± standard deviation. *p < 0.05. Non-significant differences are not shown in the graph. Group sizes were n = 3. **B** Rendering of the 3D scanned samples: Both WE43 and WE43 PEO samples show a consistent, uniform surface at 24 h, while there are cracks at 10 days (240 h)

Volume changes of the samples in response to constant contact with the medium were assessed using µCT scanning and image segmentation. The 3D images revealed that the samples’ volumes were stable until day 7 (Fig. 2A). At day 10, there was a significant decrease in volume in both WE43 and WE43 PEO samples (p < 0.0001). There was no difference in volume loss between the two groups**.** 3D renderings of the samples collected after 10 days in the medium revealed the presence of cracks along the height of the samples, which expose the bulk of the material to direct contact with the cell medium (Fig. 2B).

**Fig. 3:**
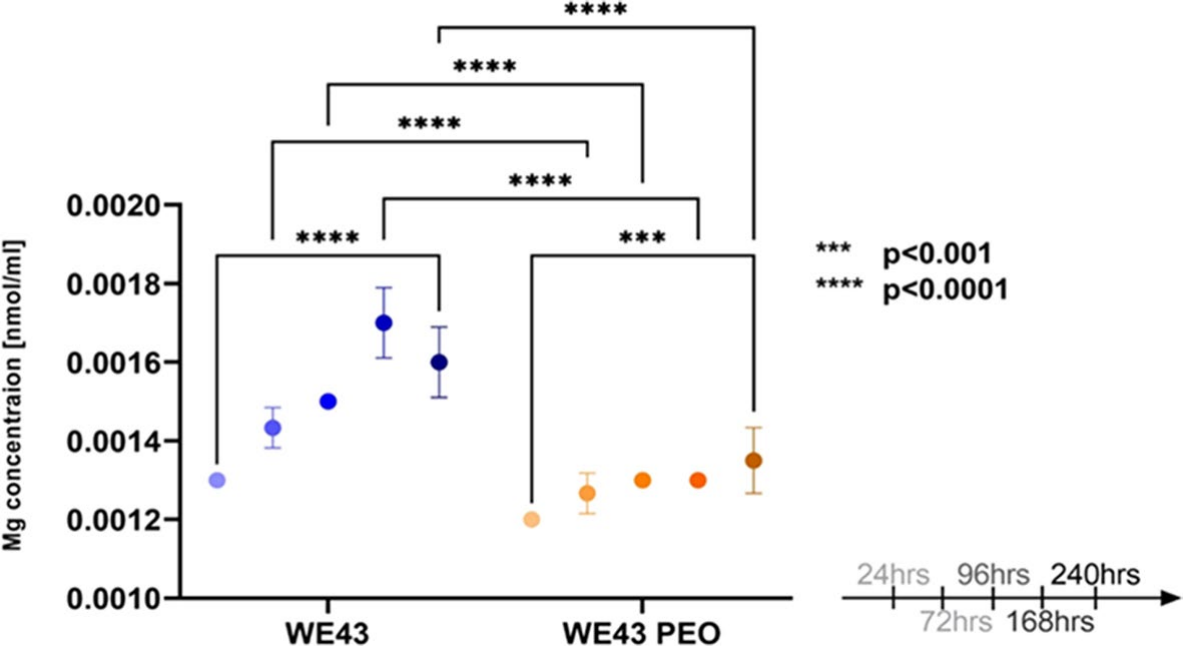
Magnesium ion release of immersed samples over time. Statistical differences are analyzed by two-way ANOVA. The error bars represendisct mean ± standard deviation. Non-significant differences are not shown in the graph. Group sizes were n = 6 for all groups

The Mg content in the supernatant was further quantified over time without medium change to verify the saturation point of Mg in the medium (Fig. 3). The highest concentration of Mg in the medium was 0.0018 nmol/ml for WE43 and 0.0014 nmol/ml for WE43 PEO samples. There was a significant difference between groups at each time point (p < 0.001—0.0001), except at 24 h, showing a higher Mg release from the non-surface-modified WE43 samples.

Although there was no difference between the groups in volume change, there was a clear contrast in the amount of Mg released. The amount of Mg in the medium was lower for the WE43 PEO samples, demonstrating the surface treatment’s effect. Only when there were apparent cracks on the WE43 PEO surface and the bulk of the sample was exposed to the medium did it display a significant increase in the amount of Mg released. Thus, the SEM images indicated a higher corrosion rate for the WE43 samples as compared to the surface-modified samples.

### Viability and osteogenic differentiation of MC3T3-E1 subclone 4 mouse cells on the surface of the Mg samples

**Fig. 4:**
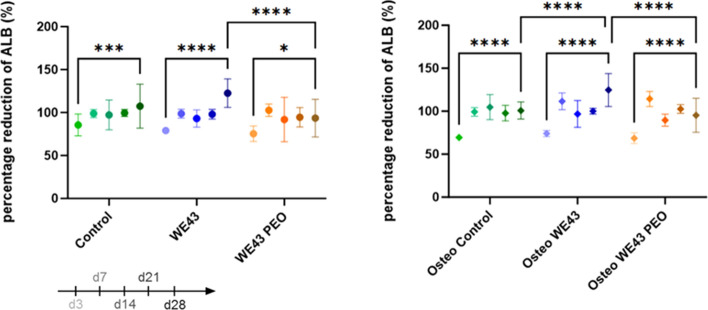
Results of the cell viability test for all groups. Statistical differences are analyzed by two-way ANOVA. The error bars represent mean ± standard deviation. *p < 0.05, ***p < 0.001, ****p < 0.0001. Non-significant differences are not shown in the graph. Group sizes were n = 6 for all groups

As shown in Fig. 4, MC3T3-E1 subgroup 4 adapted to the stress of the experiment as their viability increased significantly over time (p < 0.05). At day 28, the groups containing WE43 samples (WE43 and Osteo WE43) had significantly higher metabolic activity than the comparison group WE43 PEO samples (WE43 PEO and Osteo WE43 PEO, p < 0.0001). This data reveals that a more significant amount of Mg in the supernatant positively affected cell stress and viability. It's important to note that the Alamar Blue assay measures metabolic activity, which is often correlated with cellular health and cell viability.

**Fig. 5:**
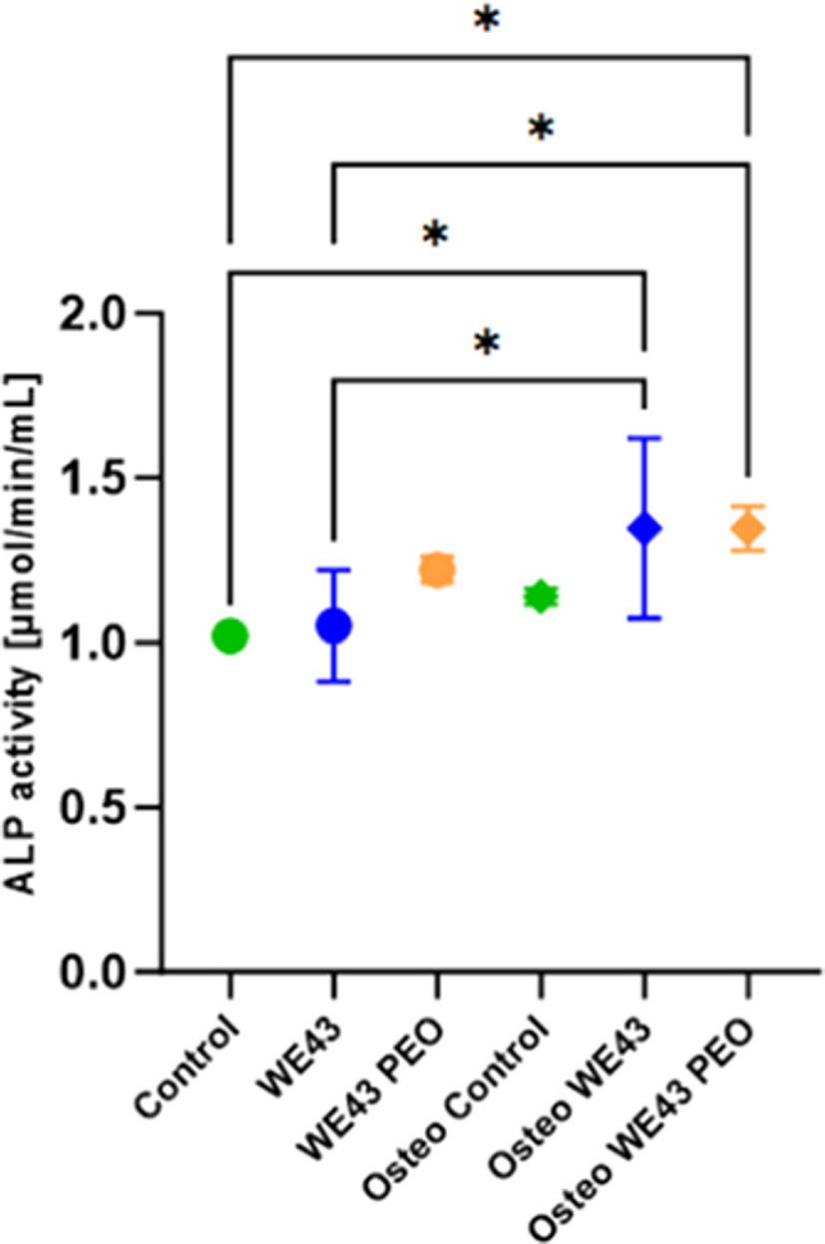
Alkaline Phosphatase Activity at d7: The combined effect of Mg and the osteogenic medium also significantly increased ALP activity. Statistical differences are analyzed by two-way ANOVA. The error bars represent mean ± standard deviation. *p < 0.05

ALP activity was measured on day 7. The MC3T3-E1 subgroup 4’s ALP activity significantly increased in the presence of Mg while cultured in an osteogenic medium (Fig. 5). The two control groups did not show any significant difference, as the Control group was cultured with regular αMEM containing some ascorbic acid, which may have contributed to their ALP production.

**Fig. 6:**
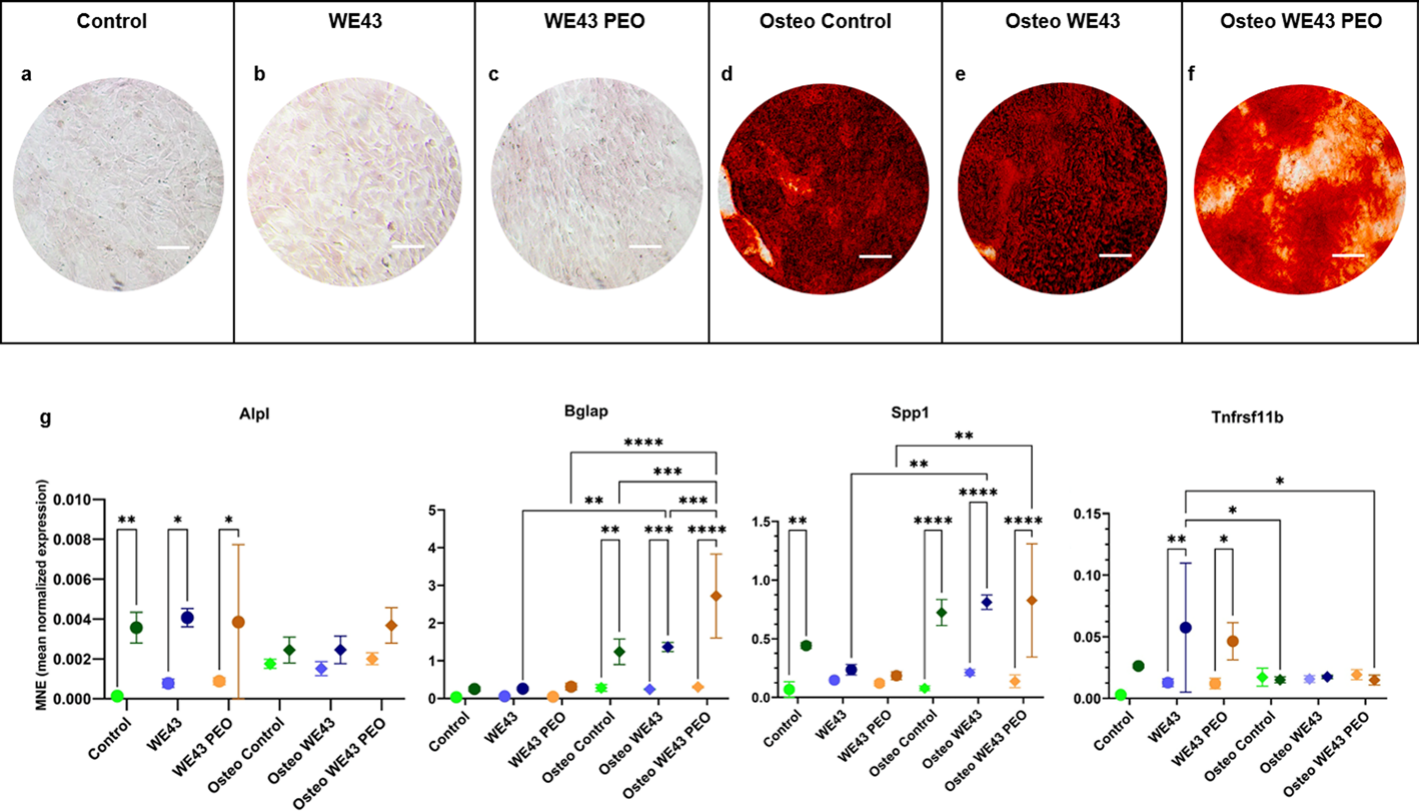
a-g: Results of the osteogenic differentiation experiments on the tested Magnesium samples. **a**–**f** Alizarin Red S staining results of the cells at day 28. The groups without osteogenic medium showed no calcium deposit. Group sizes were n = 6 for all groups. Scale bar = 100 µm. g Gene expression of osteogenesis-related genes in MC3T3-E1 subgroup 4 cells in different media and with or without Mg samples. Alpl, Bglap, Spp1, and Tnfrsf11b. Two-way ANOVA analyzes statistical differences. The error bars represent mean ± standard deviation. *p < 0.05, **p < 0.01, ***p < 0.001, ****p < 0.0001. Non-significant differences are not shown in the graph. Group sizes were n = 3 for all groups

After 28 days in culture, only the cells that received the osteogenic medium (Osteo Control, Osteo WE43, and Osteo WE43 PEO) could form calcium deposits on the extracellular matrix (Fig. 6a–f). The presence of Mg alone was not able to stimulate mineralization. The Osteo Control group, Osteo WE43, and Osteo WE43 PEO groups showed diffused mineralization throughout the culture wells. However, the staining intensity was lower in the WE43 PEO wells. Inside the wells, there were no differences in staining intensity in areas closer or further from the Mg samples.

SEM imaging was performed to verify the accumulation of organic substrate on the surface of WE43 samples. Cellular attachment and globular mineral deposition could be observed on the surface of the WE43 samples in smooth and cracked areas (Supplementary Fig. 2) as early as day 12 of the experiment. Globular accretions are characteristic of mineralizing cell cultures [15] and represent apatite minerals deposition by the cells [16]. This confirms that despite the more rapid oxidation of WE43 samples, this alloy does not disturb the dynamic early stages of osteogenesis in vitro.

Results from the RTD-PCR (Fig. 6g) revealed that WE43, regardless of the surface modification, significantly increased Alpl and Tnfrsf11b expressions over time in a non-osteogenic medium. The genes Spp1 and Bglap were upregulated considerably in osteogenic culture. The data also showed that WE43 PEO in an osteogenic environment increased the expression of Bglap compared to WE43 and control groups (p < 0.001).

### Polarization of RAW264.7 mouse macrophages in contact with WE43 samples

**Fig. 7:**
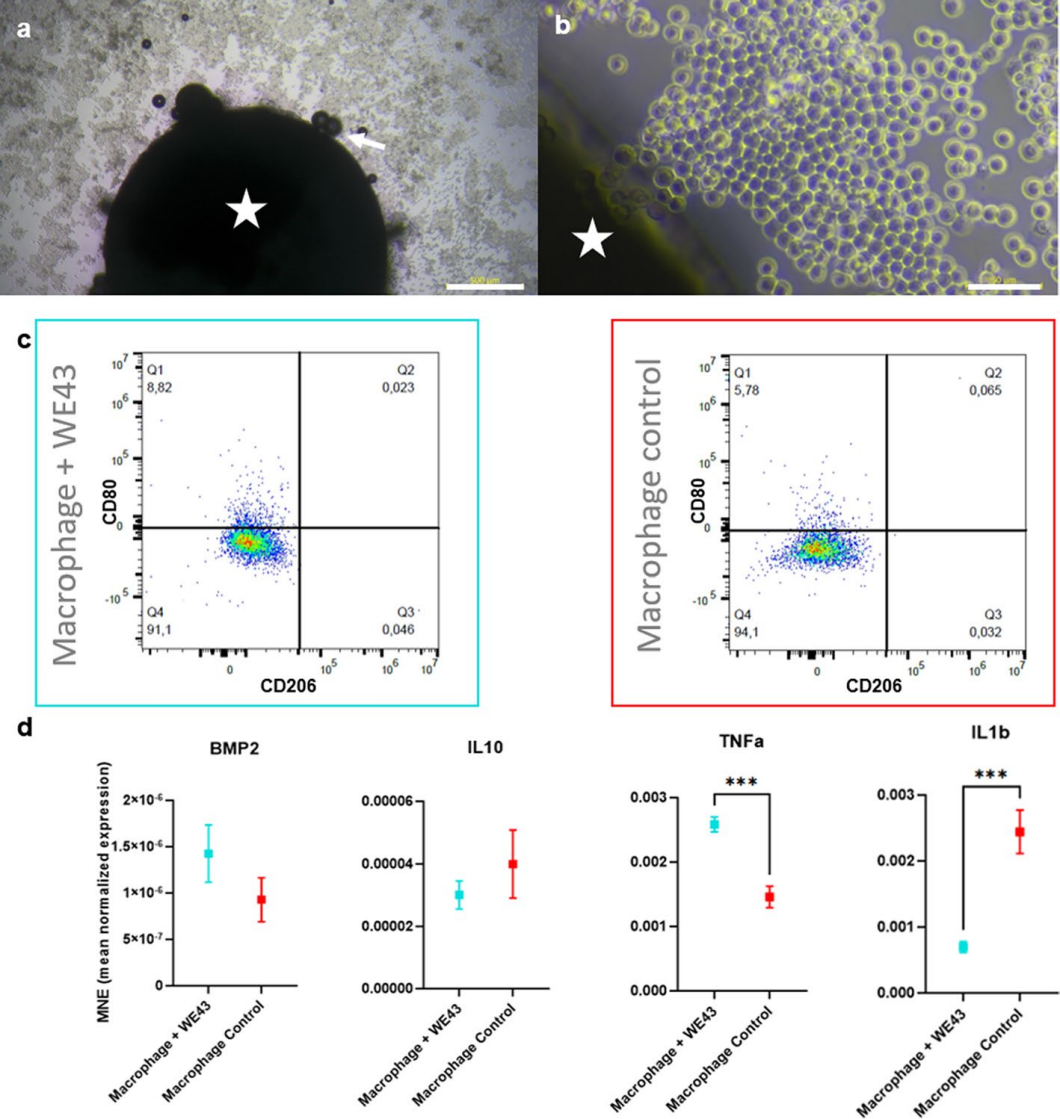
Results from the experiment with RAW264.7 cells. **a** and **b** Representative images of RAW264.7 cells at the end of the experiment taken on standard tissue culture plastic surfaces (a 4X, Scale bar: 500 µm; **b** 40X, Scale bar: 50 µm) revealing the cell morphology of the Macrophage + WE43 group (star WE43 sample, arrow bubbles released due to the oxidation of the sample and hydrogen gas release). These images illustrate cellular response and gas generation in the experimental environment, but do not depict cell adhesion on the WE43 alloy surface. **c** Expression profiles of the surface markers CD80 and CD206 from exemplary samples in the different groups were analyzed by flow cytometry. **d** Results from the RTD-PCR of the experimental cells. Statistical differences are analyzed by t-test. The error bars represent mean ± standard deviation. Non-significant differences are not shown in the graph. Group sizes were n = 6 for all groups. ***p < 0.001

Given that the PEO surface modification did not significantly enhance osteogenic differentiation in our in vitro model, we focused our investigation of the immunomodulatory effects on WE43 without PEO to assess the inherent biocompatibility of the alloy itself. RAW264.7 macrophages were cultured with WE43 for 24 h. The time frame for the experiments was chosen given the time reported for polarization of RAW 264.7 cells into M1 pro-inflammatory profile, and M2 anti-inflammatory profile [17]. Morphologically, both groups presented small-sized cells with a circular geometry (Fig. 7a and b), and there was only a minor difference in the cells´ surface marker profile (Fig. 7c). While CD206 is a marker for anti-inflammatory Macrophages M2, CD80 is a marker for pro-inflammatory Macrophages CD80. The Macrophage control group had a smaller population of cells expressing CD80 (mean 9.94%, ± 5.88%) compared to the Macrophage + WE43 group (mean 11.26%, ± 3.5%). However, the difference between groups was insignificant (p > 0.05). Furthermore, there were no identifiable cells expressing CD260 in either group. Therefore, the data suggest that the presence of WE43 did not significantly affect the polarization towards either a pro-inflammatory or anti-inflammatory phenotype.

Furthermore, to demonstrate the effect of WE43 on gene expression, some biomarkers were investigated (Fig. 7d). Tnf-α and Il-1β are typical detection markers used to investigate macrophages. In the present data, there was a significant difference in the expression of both genes by the groups. While Tnf-α was more expressed in the Macrophage + WE43 group, Il-1β was more expressed by the Macrophage control group. Other genes typically expressed by macrophages with an anti-inflammatory profile, Bmp2 and Il-10, were similarly expressed by the groups with no statistical difference.

### Osteoimmunomodulation of MC3T3-E1 subclone 4 mouse cells using the supernatant of Macrophages (RAW 267.4) cultured in contact with WE43

**Fig. 8:**
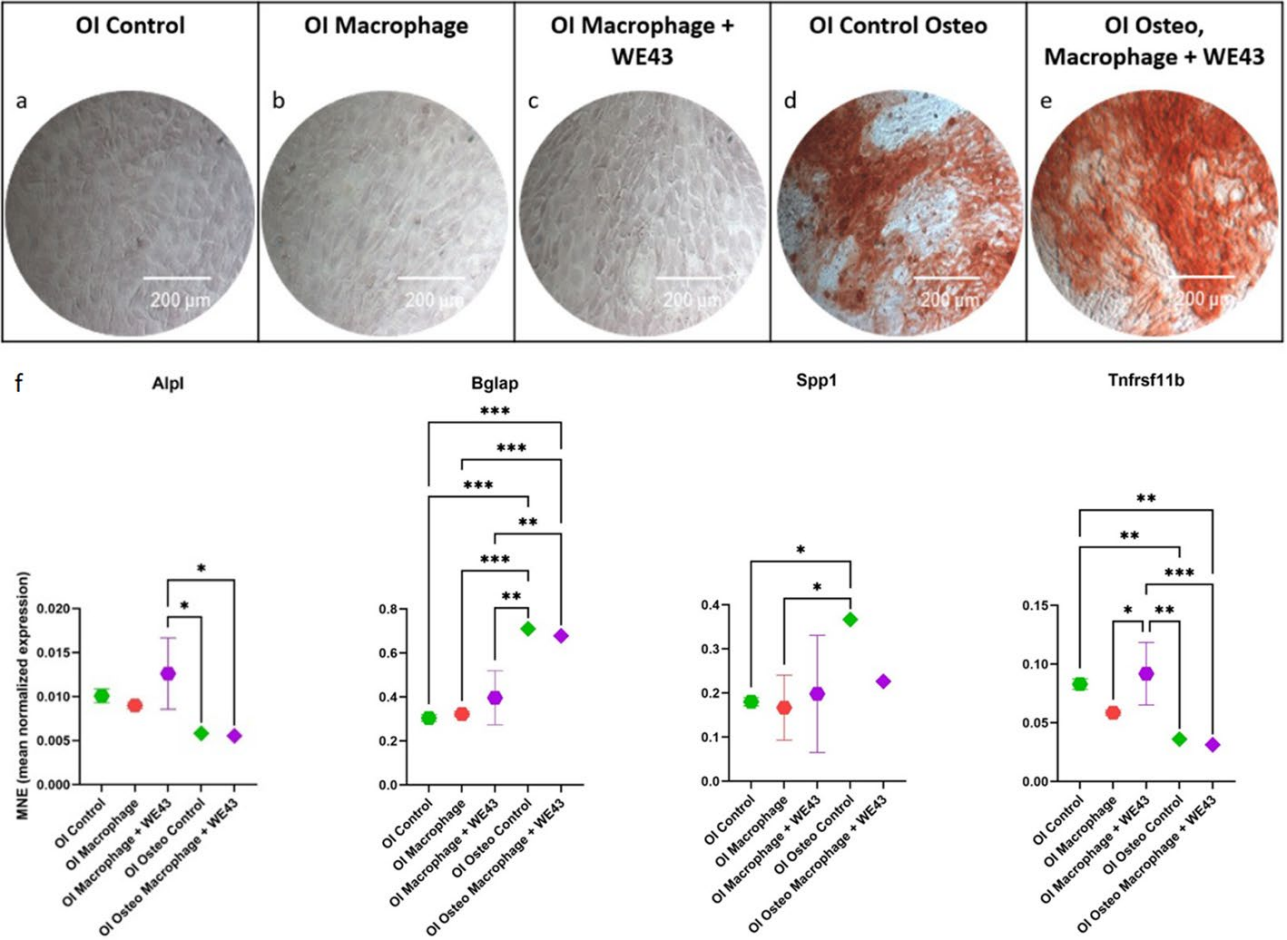
Results of the Osteoimmunomodulation experiment. **a**–**e** Alizarin Red S staining for mineralization on day 21. **f** Results from the RTD-PCR on day 21 from the osteogenesis-related expression were Alpl, Bglap, Spp1 and Tnfrs11b. Statistical differences are analyzed by two-way ANOVA. The error bars represent mean ± standard deviation. *p < 0.05, **p < 0.01, ***p < 0.001, ****p < 0.0001

Results from the osteoimmunomodulation experiment revealed that the supernatant from the Macrophages + WE43 alone did not influence the MC3T3-E1 subgroup 4 cells into mineralization (Fig. 8a–e). However, in a non-osteogenic environment, the supernatant from the Macrophage + WE43 increased the expression of Tnfrsf11b and Alpl. Furthermore, there was a decrease in the expression of Spp1 in an osteogenic environment, although not statistically significant (Fig. 8f).

## Discussion

The present results demonstrated the positive effect of WE43 on viability, Alkaline Phosphatase (ALP) expression, and osteogenic gene expression (Alpl, Tnfrsf11b, and Bglap) of osteoblasts in vitro. The presence alone of the alloy was not enough to fully stimulate osteogenesis and calcification of the extracellular matrix. However, the increased viability of osteoblasts in the presence of WE43 highlights its non-toxic and biocompatible behavior, a crucial factor for successful implant integration [18]. This biocompatibility, together with the elevated expression of ALP, an early marker of osteoblast differentiation [19] further supports the bioactive potential of WE43 in promoting bone formation [20, 21]. The upregulation of osteogenic genes such as Alpl, Tnfrsf11b, and Bglap provides evidence of WE43’s role in stimulating osteoblast differentiation. The increase in Alpl expression, which encodes ALP, confirms the enhanced osteoblast activity. The amplification of Tnfrsf11b expression, which encodes RANKL, suggests a potential role of WE43 in modulating bone remodeling through its influence on osteoclast differentiation. Furthermore, the increased expression of Bglap, a late marker of osteoblast differentiation and mineralization, underlines the alloy's ability to promote advanced stages of osteogenesis. These results highlight the potential of WE43 in the modulation of various stages of bone remodeling, such as bone resorption, bone formation, and mineralization.

Magnesium ion release was monitored for 10 days, during which concentrations in the medium reached a stable level by 168 h (Fig. 3), indicating saturation under static in vitro conditions. This early-phase exposure was considered biologically relevant, and the timeframe was therefore selected to represent the initial degradation behavior. During the 28-day cell culture period, medium was exchanged every 48–72 h to ensure continued interaction with newly released degradation products.

Since the literature on the effects of WE43 and other magnesium alloys on osteoblast gene expression is limited, it is essential to consider the broader implications of magnesium’s role in bone formation. Liu et al. [22] showed that Magnesium treatment increased the expression of PDGF-BB I in MC3T3-E1 cells, a factor known to promote both osteogenic differentiation and angiogenesis. This suggests that the positive influence of Magnesium on bone formation might be a general characteristic of this metal and may not be limited to specific alloys. The referenced study, alongside the present findings, underscores the potential of magnesium alloys like WE43 to impact bone remodeling and actively promote bone regeneration.

When comparing WE43 with and without surface modification using PEO, the data revealed that WE43 without surface modification had a more positive impact on osteoblasts’ viability, and its presence led to a starker deposit of calcium on the extracellular matrix. Concomitantly, PEO surface modification decreased the degradation rate of the alloy, which reduced the amount of Mg ions available in the medium and increased the expression of Bglap by osteoblasts in the osteogenic medium. These results indicate that the higher availability of Mg in the medium resulted in a more robust early osteogenic response to WE43. Although cells in contact with WE43 PEO increased Bglap expression, the consequences of this expression could not be ascertained by day 28.

The differences between WE43 and WE43 PEO reveal a complex interplay between degradation rate, Mg ion release, and osteogenic response. Non-surface-modified WE43 exhibits faster in vitro degradation [10], which results in a more rapid release of Mg ions. This, in turn, leads to enhanced early osteoblast activity and matrix mineralization, as shown by the greater viability and calcium deposition observed in the present study. In contrast, the PEO surface modification of WE43 PEO, while reducing the initial degradation rate and Mg release, promotes elevated Bglap expression, a marker of late-stage osteoblast differentiation. This indicates that the controlled release of Mg, as provided by the PEO coating, may be more advantageous for the later phases of osteogenesis. This may also explain the reduced early mineralization observed in the WE43 PEO group (Fig. 6), as slower degradation and lower Mg ion availability may delay initial matrix deposition, while still supporting later-stage osteogenic differentiation. This observation is further supported by the enhanced in vivo osseointegration seen with WE43 PEO by Rendenbach et al. [13] after 6 and 12 months. Furthermore, this study’s in vitro setup allowed the gas generated from the alloy degradation to dissipate without disturbing the osteogenic process, potentially leading to better results for WE43 compared to WE43 PEO.

Samples from WE43 without surface modification were used to test the alloy’s osteoimmune effect, eliminating the confounding effects of the geometry and chemical composition of the PEO surface modification. The immune response is crucial for the success of biomaterials, and a balanced reaction is essential for controlling inflammation and promoting bone regeneration. However, excessive inflammation can lead to implant failure. Thus, assessing the immune response to biomaterials is crucial to ensure implant integration. The response of RAW264.7 mouse macrophages to WE43 was tested accordingly. RAW264.7 mouse macrophages represent a well-established in vitro model for assessing immune responses to biomaterials [23]. Macrophages play a crucial role in the foreign body response, and their reaction to WE43 indicates good biocompatibility [23]. The data in the present study have shown that WE43 did not elicit a strong response from RAW264.7 mouse macrophages. However, there was an upregulation of Tnfα and a downregulation of Il1-β, both of which are pro-inflammatory cytokines that are primarily co-activated, though they can also be activated differentially [24, 25]. Depending on their environment, as they appear to have distinct regulatory mechanisms. A previous work from Negrescu et al. has shown a similar effect of another magnesium alloy (AZ31) on RAW 264.7 polarized cells, leading to a significant increase of Tnfα and a decrease of Il1ß, although not statistically significant [26]. Moreover, while there was a slight increase in CD80 expression in response to WE43, a pro-inflammatory cell marker, the overall macrophage response to WE43 appears mild. This is consistent with previous literature reporting limited inflammatory effects of magnesium alloys on immune cells in vitro. For instance, Roth et al. [27] investigated human and murine macrophages’ in vitro immune response to magnesium corrosion particles. Their findings suggest that magnesium does not significantly affect the immune function of macrophages. This study and the present findings indicate that magnesium-based biomaterials may elicit a minimal inflammatory response and are highly biocompatible.

The interaction between the immune and bone systems comprises the osteoimmune response. To investigate WE43's osteoimmunomodulatory potential, a co-culture system mimicked in vivo cellular interactions, similar to those of Hu et al. [28] and involved culturing MC3T3-E1 subgroup 4 murine osteoblasts with the supernatant collected from RAW264.7 murine macrophages activated by contact with WE43 samples. The present data revealed that the supernatant from macrophages cultured with WE43 upregulated the expression of the osteogenic markers Tnfrsf11b and Alpl in a non-osteogenic environment. Similar results have been reported in another non-osteogenic environment with a higher presence of TNFα leading to the upregulation of TNFRSF11B in melanoma cells [29], which in that instance promoted tumor cell survival [30]. Further research is necessary to verify the pathway by which macrophages’ contact with WE43 results in the upregulation of Alpl in osteoblasts, which plays a crucial role in bone formation and maintenance [31].

The present in vitro study highlights the potential of WE43 magnesium alloy as an osteoinductive and biocompatible biomaterial for bone regeneration and implant integration. This is due to its ability to support osteoblast activity, regulate osteogenic gene expression, and exhibit a mild, anti-inflammatory immune response, modulating both osteogenesis and the immune response. While uncoated WE43 favors early-stage osteogenesis by promoting osteoblast activity and matrix mineralization through rapid magnesium release, the controlled Mg release and reduction of gas formation from WE43 PEO supports advanced differentiation and prolonged osseointegration [11]. It is paramount to remember that although WE43 presents a more pronounced osteogenic effect, especially in the early stages of bone formation, its gas formation can affect the clinical outcome, leading in worst-case scenarios to bone loss.

These findings highlight the importance of optimizing degradation rates and immune responses for effective bone healing. Further research, particularly in vivo studies, will be essential to assess the clinically relevant osteoimmune response to WE43 for long-term bone regeneration and successful implant integration.

## Limitations

This study was designed to isolate and investigate the early biological response to the WE43 alloy under controlled in vitro conditions. Certain aspects were intentionally not addressed due to the inherent limitations of in vitro models.

No direct morphological imaging (e.g., cytoskeletal staining) was performed to confirm cell adhesion, particularly for the WE43 PEO group and at later time points. SEM analysis at day 12 offered indirect evidence but cannot fully substitute high-resolution cell-level visualization.
Degradation and ion release were assessed over 10 days, which was sufficient to characterize the early ionic environment. A direct correlation with cellular behavior over 28 days is limited, but longer-term degradation profiles are best evaluated in vivo.
The immunomodulatory response was investigated only for the uncoated WE43 alloy to establish a clear baseline. A comparative immune analysis of the PEO-coated alloy was beyond the scope of this study and should be addressed in future work.
Some microscopy images, including those shown in Fig. 7, were taken on standard tissue culture plastic and do not represent direct cell-material interaction on the magnesium samples.
Mineralization was assessed qualitatively based on Alizarin Red S staining. While group differences were consistent and supported by gene expression data, quantitative methods would improve objectivity in future studies.
(6) The in vitro culture period was limited to 28 days for osteoblasts and 24 h for macrophages, reflecting standard biological windows: longer culture durations compromise model validity due to overconfluence (osteoblasts) or loss of polarization (macrophages).

Overall, this study deliberately focused on the early-phase response to WE43 and WE43 PEO. Many of the open questions, e.g. long-term degradation behavior require in vivo models and were intentionally excluded from the current in vitro design.

## Methodology

### Material Characterization:

**Fig. 9:**
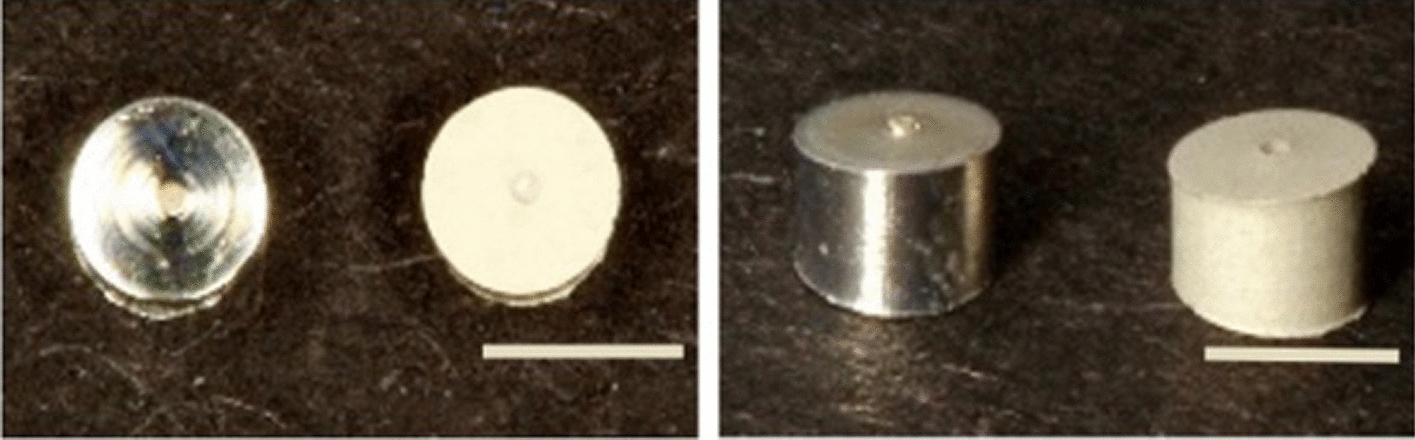
WE43 specimens without (left) and with PEO surface modification (right) in an upper (**a**) and lateral view (**b**). Scale bar = 2 mm

All samples (n = 126) were based on chill-casted and extruded Mg-Y-RE-Zr alloy WE43MEO (Meotec GmbH, Aachen, Germany) with an elemental composition of 1.4–4.2% Y, 2.5–3.5% Nd, < 1% (Al, Fe, Cu, Ni, Mn, Zn, Zr) and balance Mg (in wt-%). The samples were Swiss turned into cylinders of Ø 1.8 mm x L 1.1 mm and 1.4 mm (KLS Martin SE & Co. KG, Tuttlingen, Germany) in a trapezoidal shape (Fig. 9). Part of the samples (n = 36) were additionally surface modified using plasma-electrolytic oxidation (PEO) using a phosphate-based electrolyte (Kermasorb^®^). To reach the specific modification thickness, processing time, and electrical parameters of the bi-polar pulsed rectifier setup (M-PEO A1, Meotec GmbH) were Galvano-statically adjusted starting at 10 A/m2.

#### SEM + EDX analysis of both Mg implant types

The samples were examined with a Scanning electron microscope (Jeol, JCM 6000) coupled with an energy-dispersive X-ray spectroscopy (EDX) system at 15 kV. The surface components were assessed.

#### Implant degradation and magnesium release

For the analysis of the in vitro degradation and Mg release within the used medium for cell culture, Mg samples (WE43 and WE43 PEO, n = 6 for each time point) were placed in the Culture Medium for 24 h, 72 h, 96 h, 168 h, and 240 h, without medium changes. The magnesium amount released within the medium was analyzed with a Magnesium Detection Kit (Abcam, catalog number ab102506). After that µCT scans of the samples were performed (SkyScan 1172, Brucker, running at 80 kV tube voltage and 124 µA tube electric current, with a scan resolution of 5 µm and exposure time of 1000 ms) to assess the degradation of the samples over time through volume loss. Images were reconstructed using NRecon (Brucker), and volume was analyzed using Fiji (ImageJ Version 1.52f.)

### Viability and osteogenic activity of MC3T3-E1 subclone 4 mouse cells on the surface of WE43 samples

#### Cell culture and medium preparation

MC3T3-E1 subclone 4 cells (ATCC No: CRL − 2593^™^) were chosen as a well-established osteogenesis model since they have shown high differentiation and mineralization potential after growth in the presence of ascorbic acid [32].

The cells were cultured with alpha-minimal essential medium (α-MEM) (ATCC: Gibco^™^, USA, catalog number A1049001) supplemented with 10% fetal bovine serum (FBS) (Biochrom, USA, catalog number S 0115), 1% penicillin–streptomycin solution (Bio&Sell, USA, catalog number BS.A2213).

For osteogenic differentiation, 50 µg/mL ascorbic acid (Gibco^™^, catalog number 32571–028) and 10mM beta-glycerol phosphate (Sigma, catalog number 50020) were added to the medium. Wells, without any added ascorbic acid, were kept to test the osteogenic potential of the different Mg samples on the cells. In total, there were six tested conditions:

1. Control: Cells cultured without osteogenic induction
2. WE43: Cells seeded on WE43 samples.
3. WE43 PEO: Cells seeded on WE43 PEO samples.
4. Osteo Control: Cells cultured with osteogenic induction.
5. Osteo WE43: Cells seeded on WE43 samples and with osteogenic induction.
6. Osteo WE43 PEO: Cells seeded on WE43 PEO samples and with osteogenic induction.

The cells were seeded into 24 well plates with a seeding density of 3.500 cells/cm^2^ and cultured for 28 days. Several tests were performed with each well at different time points:

- Alamar Blue for cell viability on days 3, 7, 14, 21 and 28
- Alkaline Phosphatase (ALP) Assay for ALP activity on day 7
- Alizarin Red S Staining for mineralization on day 28
- Real-time detection polymerase chain reaction (RTD-PCR) for osteogenic gene expression on days 7 and 28

##### Cell viability test

Cell viability was assessed using the alamarBlue^™^ Cell Viability Reagent (Invitrogen^™^, catalog number DAL1025) on days 3, 7, 14, 21, and 28, according to the manufacturer’s instructions. Measurements were performed using a microplate reader (Tecan Infinite® 200 Pro). Background normalization was performed by subtracting blank values.

##### Alkaline phosphatase (ALP) assay to determine ALP activity

Alkaline Phosphatase is an enzyme that represents an early marker during osteogenesis [33]. On day 7, the supernatant of each group was detected for ALP activity using the ALP Assay Kit (Abcam, catalog number ab83369). Measurements were performed using a microplate reader (Tecan Infinite® 200 Pro). Background normalization was performed by subtracting blank values.

##### Alizarin red s staining for mineralization on day 28

Staining with the Alizarin Red S dye (Sigma Aldrich, catalog number 2003999), which stains calcium deposits, was performed on day 28 to detect cell mineralization. The medium was removed for the staining, and the cells were washed three times with 100 µL PBS. The cells were then fixed with 4% Paraformaldehyde in PBS (PFA, Santa Cruz Biotechnology, catalog number SC-281692) for 15 min and rinsed with deionized water thrice. The cells were then stained with 0,2 mL 40 mM Alizarin Red S solution, incubated under slight movement for 30 min, and then rinsed well with deionized water five times. To determine any mineral deposits, the wells were imaged under a transmission light microscope (Axiovert 25, Zeiss).

##### Real-time detection polymerase chain reaction (RTD-PCR) for gene expression

RTD-PCR was used to evaluate the expression of osteogenesis-related genes (Supplementary Table 1) on days 7 and 28. According to the manufacturer’s protocol, total RNA was isolated from the cells using the RNeasy mini kit (Qiagen, Hilden, Germany). Then, the RNA quantity and quality were evaluated with the NanoDrop ND1000 Spectrophotometer (Thermo Fischer Scientific, Waltham, MA, USA).

After that, the RNA was transcribed into cDNA (complementary DNA) using the QuantiTect Reverse Transcription Kit (Qiagen, Hilden, Germany) according to the manufacturer’s protocol. Depending on RNA availability, 125–1000 ng of total RNA was used. In all cases, the final cDNA volume was adjusted to 20 µL, ensuring uniform conditions for subsequent qPCR analysis despite differences in the initial RNA quantity.

The expressions of the osteogenesis-related genes, including alkaline phosphatase (Alpl), Osteocalcin (Bglap), secreted phosphoprotein 1 (Osteopontin, Spp1), tumor necrosis factor receptor superfamily member 11b (Osteoprotegerin, Tnfrsf11b) and the housekeeping gene β-Actin (Actb) (all primers derived from Thermo Fischer Scientific, Waltham, MA, USA) were quantified with a StepOnePlus thermocycler (Applied Biosystems (ABI), Foster City, CA, USA) using StepOnePlus software 2.3 (ABI) on the SYBR Green PCR Master Mix (ABI, catalog number 4309155) according to the manufacturers instruction. Relative gene expression levels were normalized to the housekeeping gene β-Actin. The genes' mean normalized expressions (NME) were calculated by the modified deltaCt method [34]. Details on the methods are in the Supplementary Information.

### Polarization of RAW264.7 mouse macrophages in contact with WE43 samples

#### Cell culture and medium preparation

To evaluate the innate immune response potential of WE43 alloy, RAW264.7 murine-derived macrophage-like cells (ATCC, catalog number: TIB-71) were cultured for 24 h at a seeding density of 10.000/cm^2^ with and without contact with WE43 samples. The RAW264.7 cells were used in an unpolarized (M0) state to assess macrophages’ direct, unconditioned response to the WE43 alloy.

The culture medium for the RAW264.7 cells consisted of Dulbecco’s Modified Eagle’s Medium (DMEM high glucose, Gibco™, catalog number 80514904) supplemented with 10% fetal bovine serum (FBS) (Biochrom, USA, catalog number S 0115), 1% penicillin–streptomycin solution (Bio&Sell, USA, catalog number BS.A2213). The cells were seeded into 24-well plates.

For the further co-culture experiments (2.4.), the cells´ supernatant was collected and stored at -20°C.

#### Fluorescence-activated cell sorting (FACS) analysis of cell surface markers and phenotyping

After morphological assessment, the cells were detached with Accutase (BioLegend, catalog number 423201). They were then resuspended in staining buffer (PBS + 2% FBS) at a concentration of 5 × 10^6^ cells/ml. The cells were then incubated with the antibodies for at least 20 min at 4°C in the dark and fixated.

A full-spectrum flow cytometer, Aurora (CytekTM), was used for the fluorescence-activated cell sorting (FACS) of the RAW264.7 cells. To identify the cells' polarization, CD80 (16-10A1 BioLegend, San Diego, CA, USA) for M1 macrophages and CD206 (C068C2 BioLegend, San Diego, CA, USA) for M2 polarization were used. The cells were gated for size, single cells, live cells (LIVE/DEAD^™^ Fixable Blue Dead Cell Stain Kit, Invitrogen), and CD45 expressing cells (I3/2.3 BioLegend, San Diego, CA, USA) using FlowJo™ v10.8 Software (BD Life Sciences). The generated data from two experiments, which comprised 6 samples from each group, were analyzed.

#### Real-time detection polymerase chain reaction (RTD-PCR) for gene expression after 20 h

RTD-PCR, the same technique already described, was used to evaluate the expression of immunomodulatory-related genes (Supplementary Table 1) after 24 h.

The expressions of the immunomodulatory-related genes, including bone morphogenic protein type 2 (Bmp2), interleukin 10 (Il-10), tumor necrosis factor α (Tnf-α), interleukin 1β (Il-1β), and the housekeeping gene β-Actin (Actb) (all primers derived from Thermo Fischer Scientific, Waltham, MA, USA) were quantified.

### Osteoimmunomodulation co-culture of MC3T3-E1 subclone 4 mouse cells using the supernatant of Macrophages (RAW 267.4) cultured in contact with WE43

To investigate the early osteoimmunomodulatory effect of WE43 samples, the supernatant of RAW264.7 cells activated or not by contact with WE43 for 20 hs was added to MC3T3-E1 subclone 4 culture. MC3T3-E1 subclone 4 were cultured with:

1. OI Control: Growth medium.
2. OI Macrophage: Growth medium and Macrophage (RAW 264.7) supernatant in a 1:1 mix.
3. OI Macrophage + WE43: Growth medium and WE43 activated Macrophage (RAW 264.7) supernatant in a 1:1 mix.
4. OI Osteo Control: Osteogenic medium.
5. OI Osteo Macrophage + WE43: Osteogenic medium and WE43 activated Macrophage (RAW 264.7) supernatant in a 1:1 mix.

The cells were seeded into a 96-well plate and cultured for 21 days. After that, the samples were stained with Alizarin Red S or used for RT-PCR to assess the expression of osteogenesis-related genes (Alpl, Bglap, Spp1, and Tnfrsf11b).

### Statistical analysis

All data were tabulated and analyzed using GraphPad Prism 10 (GraphPad Software Inc., San Diego, CA, USA). Data are presented as mean ± standard deviation. Statistical differences were analyzed by two-way ANOVA with the Tukey post hoc test. The level of significance was set at p values of p < 0.05 (*), p < 0.01 (**), p < 0.001 (***), p < 0.0001 (****).

## Supplementary Information

Supplementary material 1.

## Data Availability

The data generated and analyzed during the current study are available from the corresponding author on reasonable request.
