# Supplementary material for: In vitro study on the osteoimmunological potential of magnesium implants (WE43MEO)
Source: Biomed Eng Online. 2025 Jul 15;24:90. doi: 10.1186/s12938-025-01413-5 (PMC12265262; doi:10.1186/s12938-025-01413-5)
Supplement: Supplementary file 1 — Supplementary material 1. [file 12938_2025_1413_MOESM1_ESM.docx]

**Supporting Information:**

**1. Real time detection – polymerase chain reaction (RTD-PCR)**

RTD-PCR was used to evaluate the expression of osteogenesis-related genes (Supplementary Table 1) on days 7 and 28. To do so, total RNA was isolated from the cells using the RNeasy mini kit (Qiagen, Hilden, Germany) according to the manufacturer's protocol. Then, the RNA quantity and quality were evaluated with the NanoDrop ND1000 Spectrophotometer (Thermo Fischer Scientific, Waltham, MA, USA).

After that, the RNA was transcribed into cDNA (complementary DNA) using the QuantiTect Reverse Transcription Kit (Qiagen, Hilden, Germany) according to the manufacturer's protocol. Depending on RNA availability, 125-1000 ng of total RNA were used. In all cases, the final cDNA volume was adjusted to 20µl. The final cDNA volume was consistently adjusted to 20µl, ensuring uniform conditions for subsequent qPCR analysis despite differences in the initial RNA quantity.

The expressions of the osteogenesis-related genes, including alkaline phosphatase (Alpl), Osteocalcin (Bglap), secreted phosphoprotein 1 (Osteopontin, Spp1), tumor necrosis factor receptor superfamily member 11b (Osteoprotegerin, Tnfrsf11b) and the housekeeping gene β-Actin (Actb), were determined.

Primer sets for the target genes Alpl, Bglap, Spp1, Tnfrsf11b, and the housekeeping gene Actb were designed manually, considering parameters such as melting temperature, GC content, primer length, and potential secondary structures. Melting temperature values were kept between 55-60°C for all primer pairs. GC content was maintained between 40-60%. Primers were designed to be 18-24 bases long. Potential secondary structures were assessed by keeping the parameters “self-complementarity” and “self 3′-complementarity” as low as possible.

Primer efficiency was evaluated by generating a standard curve using a serial dilution of cDNA in a standard RTD-PCR run for every primer pair. Log quantity values were plottet against the average Ct values followed by generating a trendline and regression equation with Excel. The efficiency for each primer pair was calculated using the resulting slope value with the following formula: 10^(-1/slope value), reference range (1,80-2,10), whereby 2,00 indicates perfect doubling of PCR product per cycle. The determined primer efficiencies were as follows: Alpl (1,91), Bglap (1,88), Spp1 (1,91), Tnfrsf11b (1,8), and Actb (1,98).

All primers were obtained from Thermo Fisher Scientific, with a final primer concentration of 400 nM. The final cDNA concentration was 10 ng/µl (1 ng per reaction). RT-qPCR reactions were prepared using SYBR Green PCR Master Mix (2X concentration), containing SYBR Green I Dye, AmpliTaq Gold DNA Polymerase, dNTPs, Passive Reference, and optimized buffer components, with final master mix component concentrations prepared in a total reaction volume of 10 µl.

The following cycling conditions were used: an initial activation phase of 95°C for 10 minutes, followed by 40 cycles of denaturation at 95°C for 15 seconds and combined annealing and extension at 60°C for 1 minute per cycle. The run concluded with a final cooling phase at 8°C for 10 minutes. Three blanks (NTC) were included in each run.

RTD-PCR was performed using a StepOnePlus thermocycler (Applied Biosystems (ABI), Foster City, CA, USA) using StepOnePlus software 2.3 (ABI) on the SYBR Green PCR Master Mix (ABI, catalog number 4309155) according to the manufacturers instruction. Relative gene expression levels were normalized to the housekeeping gene β-Actin. The genes' mean normalized expressions (NME) were calculated by the modified deltaCt-Method [14].

| **Gene** | **Gene name** | **Primer sequence** | **Accession no.** | **Product size (bp)** | **Efficiency** |
| --- | --- | --- | --- | --- | --- |
| **Actb** | β-Actin | F: CATTGCTGACAGGATGCAGAAGG R: TGCTGGAAGGTGGACAGTGAGG | NM_007393.5 | 138 | 1,98 |
| **Alpl** | Alkaline phosphatase | F: ACTCAGGGCAATGAGGTCAC R: CTGGTGGCATCTCGTTATCC | NM_007431.3 | 160 | 1,91 |
| **Bglap (OC)** | Bone γ-carboxylglutamic acid-containing protein (Osteocalcin) | F: CCTGGCTGCGCTCTGTCT R: TGCTTGGACATGAAGGCTTTG | NM_007541.3 | 81 | 1,88 |
| **Bmp2** | Bone morphogenic protein type 2 | F: AACACCGTGCGCAGCTTCCATC R: CGGAAGATCTGGAGTTCTGCAG | NM_007553.3 | 143 | 2,02 |
| **Il-1β** | Interleukin 1β | F: TGGACCTTCCAGGATGAGGACA R: GTTCATCTCGGAGCCTGTAGTG | NM_008361.4 | 148 | 1,96 |
| **Il-10** | Interleukin 10 | F: CGGGAAGACAATAACTGCACCC R: CGGTTAGCAGTATGTTGTCCAGC | NM_010548.2 | 130 | 1,93 |
| **Spp1 (OP)** | Secreted phosphoprotein 1 (Osteopontin) | F: TGGTGCCTGACCCATCTCA R: TTCATTGGAATTGCTTGGAAGA | NM_009263.3 | 110 | 1,91 |
| **Tnf-α** | Tumor necrosis factor α | F: GGTGCCTATGTCTCAGCCTCTT R: GCCATAGAACTGATGAGAGGGAG | NM_001278601.1 | 139 | 2,04 |
| **Tnfrsf11b (Opg)** | Tumor necrosis factor receptor superfamily member 11b (Osteoprotegerin) | F: CCTGAGGTTTCCCGAGGACC R: TACTTTGGAGGAAGGGTTTCCTGG | NM_008764.3 | 106 | 1,80 |

**Supplementary table 1:** Mouse specific primers used for RTD-PCR in this study. All primers were obtained from Thermo Fisher Scientific, Waltham, MA, USA.
RTD-PCR: real time detection – polymerase chain reaction. bp: base pairs

**2. Energy Dispersive X-ray Spectroscopy**

Both implant samples, WE43 and WE43 PEO, were prepared and then analyzed for their elemental composition using Energy Dispersive X-ray Spectroscopy (EDX) imbedded in a Scanning electron microscope (Jeol, Jcm 6000). Each sample was removed from their sterile packaging using sterilized plastic tweezer. The samples were then securely mounted on aluminum stubs with conductive carbon tape to ensure stable and accurate signal detection during the EDX analysis. For the elemental composition analysis, spectra were collected on multiple areas over the sample surfaces to ensure consistent and representative data.

**Supplementary figure 1:** EDX analysis of WE43 and WE43 PEO. The WE43 samples presented a uniform surface with 100% Mg. Analysis of the WE43 PEO samples revealed the presence of carbon (8.52%), oxygen (26.91%), magnesium (28.79%), aluminum (1.89%), and phosphorus (33.89%). The high amount of Oxygen can be counted for the oxidation of the surface and the presence of the other elements in an oxidized state (as shown by Kopp et al. [15]). Furthermore, the amount of Al might be due to the construction of the chamber of the SEM and holders made with this element.

**3. Scanning Electron Microscopy (SEM) of WE43 in osteogenic medium post 12 days**

To examine the presence of calcium deposition on the surface of the Mg samples, MC3T3-E1 cells were cultured on WE43 samples with the osteogenic medium for 14 days. The samples were then processed for imaging. The MC3T3-E1 cells were fixed with primary fixative containing 2.5% glutaraldehyde in 0.1 M phosphate buffer for 30-60 minutes, then washed three times with 70% alcohol for 2 minutes each and dehydrated with a graded series of ethanol dilutions: first in an 80% solution (1x5min), then in a 90% solution (1x5min), followed by a 95% solution (1x5min), and finally in 100% ethanol (3×7min). The samples were dried using the Leica EM CPD300 critical point dryer. The samples were then placed on aluminum stubs with conductive carbon tape to ensure stability during imaging. Imaging was performed using a scanning electron microscope (Jeol, JCM-6000) to observe the surface structure and calcium deposits. High-vacuum imaging with optimized voltage allowed detailed topographical observation revealing mineralization patterns and cell attachment.


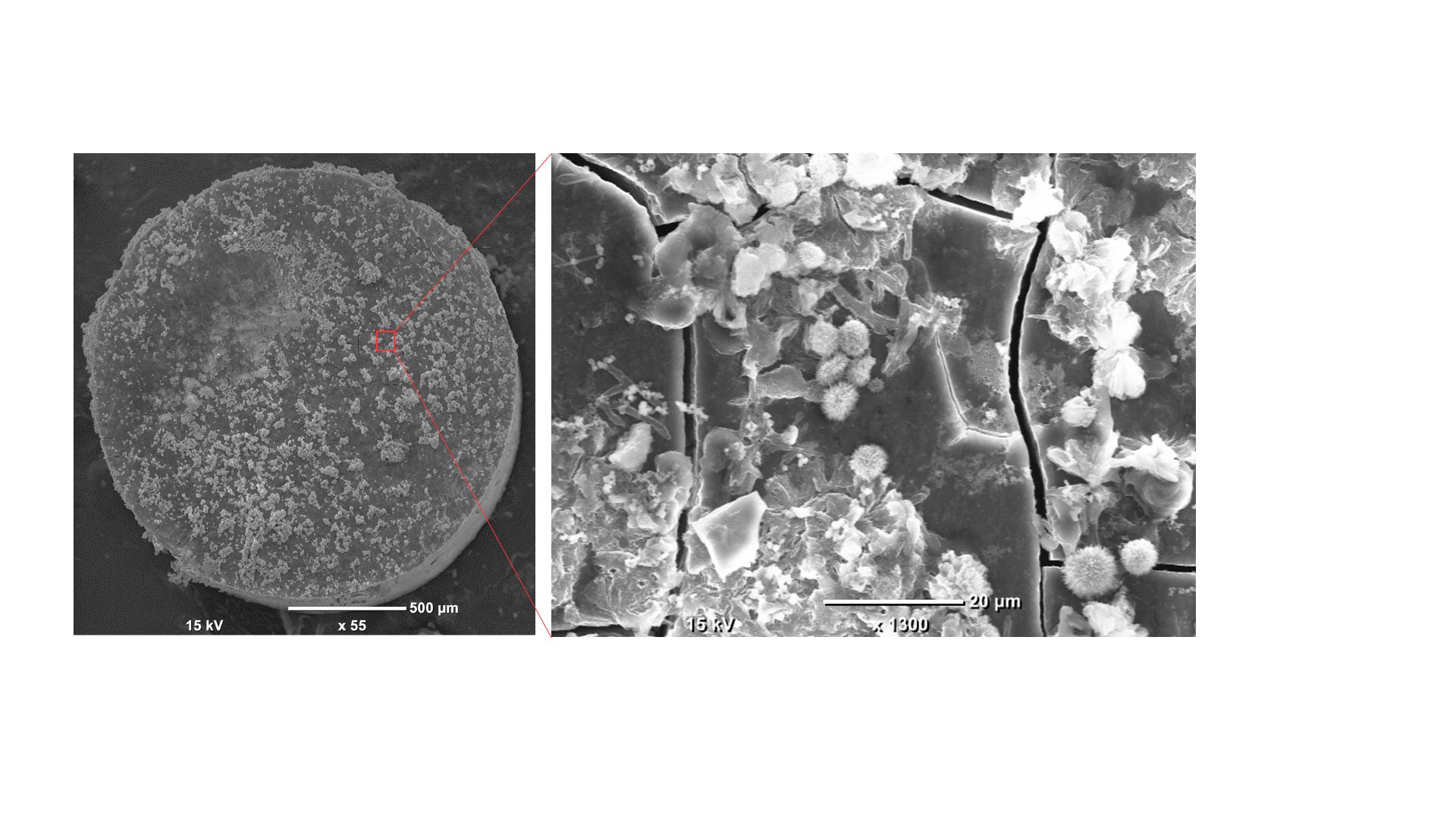


**Supplementary figure 2:** SEM of WE43 in osteogenic medium post 12 days. SEM analysis revealed early globular mineralization (*) as small spherical foci along with attached cells (arrows) on the surface of the WE43 samples.
